# Neuropsychological rehabilitation in a patient with ruptured anterior communicating artery aneurysm: 48 month outcomes

**DOI:** 10.1590/S1980-57642008DN10400014

**Published:** 2007

**Authors:** Silvia A. Prado Bolognani, Priscila Covre, Daniella Landucci-Moreira, Thiago S. Rivero, Sonia Maria Dozzi Brucki, Orlando Francisco Amodeo Bueno

**Affiliations:** 1 Neuropsychologist. Department of Psychobiology, Federal University of Sao Paulo (UNIFESP), Brazil.; 2 MsC, Psychologist. Department of Psychobiology, Federal University of Sao Paulo (UNIFESP), Brazil.; 3 Psychologist. Department of Psychobiology, Federal University of Sao Paulo (UNIFESP), Brazil.; 4 Graduate student, Psychologist. Department of Psychobiology, Federal University of Sao Paulo (UNIFESP), Brazil.; 5 MD, PhD. Department of Psychobiology, Federal University of Sao Paulo (UNIFESP), Brazil.; 6 MsC, PhD, Head of Department, Department of Psychobiology, Federal University of Sao Paulo (UNIFESP), Brazil.

**Keywords:** neuropsychological rehabilitation, executive functions, amnesia, behavioral disturbances, activities of daily living, reabilitação neuropsicológica, funções executivas, amnésia, distúrbios comportamentais, atividades da vida diária

## Abstract

The neurobehavioral impairments associated with aneurysms of the anterior communicating artery (ACoA) are severe amnesia, executive problems and personality changes. Although most patients achieve a favorable neurological outcome, those cognitive deficits usually prevent return to previous activities and levels of social integration.

**Objectives:**

To report the outcomes of a neuropsychological and behavioral intervention in a 55 year-old man with very severe memory and executive dysfunctions following ACoA aneurysm rupture.

**Methods:**

Neuropsychological intervention focused in functional adjustment in everyday life was used, including individual sessions with the patient, discussion sessions with caregivers and also work with patient at home, aiming generalization of the rehabilitation strategies. Neuropsychological and functional assessments were conducted pre and post intervention.

**Results:**

Important improvements were seeing in behavior and daily living performance after treatment.

**Conclusions:**

A neuropsychological rehabilitation approach focused on goals based on the family and caregivers necessities is an efficient manner in which to carry out cognitive rehabilitation in severe cases. The importance of a supportive family should be stressed.

## Abstract (pt)

Os prejuízos neuro-comportamentais associados a aneurismas de artéria comunicante anterior (ACoA) são amnésia severa, dificuldades executivas e alterações de personalidade. Embora muitos pacientes alcancem uma recuperação neurológica favorável, os prejuízos cognitivos acabam impedindo o retorno às atividades prévias e ao mesmo nível de integração social.

**Objetivos:**

Relatar os resultados de uma intervenção neuropsicológica e comportamental junto a um homem de 55 anos, com dificuldades severas de memória e funções executivas secundárias a ruptura de aneurisma de ACoA.

**Métodos:**

Foi empregada intervenção neuropsicológica com foco na adaptação funcional na vida diária. Foram realizadas sessões individuais com o paciente, sessões de discussão com cuidadores, além de trabalho com o paciente em casa, visando a generalização das estratégias de reabilitação. Foram realizadas avaliações neuropsicológica e funcional pré e pós-intervenção.

**Resultados:**

Foram observadas melhoras importantes no comportamento e no desempenho da vida diária após o tratamento.

**Conclusões:**

reabilitação neuropsicológica com foco nas necessidades dos familiares e cuidadores revelou-se eficiente maneira de realizar a reabilitação neuropsicológica em casos graves. A importância do apoio da família deve ser ressaltada.

One of the most common arterial locations for aneurysms is the anterior communicating artery (ACoA).^1^ ACoA aneurysm rupture is consistently associated with executive and memory dysfunctions,^2-4^ given that the frontal lobes and basal forebrain are the most frequently injured sites.^3,5,6^ The cluster of neurobehavioral impairments associated with ACoA aneurysm has been referred to as the “ACoA syndrome”, and consists of severe amnesia, confabulation along with personality changes.^7^ In most cases, individuals with ACoA aneurysm achieve a favorable neurological outcome, however the presence of cognitive deficits is associated with inability to return to work and resume previous levels of social integration.^8^ For those patients with severe cognitive impairment that return to their home environment, adjustment to functional life can be difficult. Memory and executive deficits may disrupt the performing of basic daily living activities and even normal interaction at home, thus generating greater burden for family and carers. Literature on neuropsychological rehabilitation has provided sufficient evidence that the most efficient approach for those cases is goal-oriented intervention, rather than providing only “general stimulation” with cognitive exercises.^9^ Thus, rehabilitation should focus on functional adjustment in everyday life, thereby enabling family and patient to cope better with the new situation after the injury. The purpose of this paper was to report the outcomes of neuropsychological and behavioral intervention in a patient with very severe memory and executive dysfunctions following ACoA aneurysm rupture.

## Methods

### Patient

MM, 55 years’ old, right-handed, graduated in Economics and holding a post-graduate degree in Business, had a cerebral hemorrhage caused by a ruptured ACoA aneurysm, with cardiopulmonary arrest, approximately four years earlier. He was submitted to a successful aneurysm embolization. Several days later, hydrocephalus was detected and he underwent ventricle peritoneal derivation.

At the time of referral for rehabilitation, 10 months after the injury, he showed persistent cognitive and behavioral impairments and very mild left hemiparesis. His medications at the evaluation were galantamine (8 mg/d); topiramate (75 mg/d), amitriptyline (12.5 mg/d), carbamazepine (100 mg/d) and memantine (10 mg/d). These medications were tapered slowly, while galantamine was gradually increased up to a dose of 24 mg/d. His latest CT scan showed a metallic artifact on ACoA topography, and bilateral frontal and right internal capsule gliosis.

Data from Neuropsychological Assessments are reported in Table 1 while Functional Assessments are shown in Table 2. Functional assessment was accomplished through clinical interviews with wife and caregiver, and also behavioural observation at the clinic. Based on findings from the initial assessments, the team and family began devising rehabilitation goals and strategies of achievement, which were reviewed and improved as MM progressed. Outcome was evaluated 3 years later.

**Table 1:** Neuropsychological results before and after rehabilitation.

Cognitive Functions/Tests	Month 10 (Before intervention)	Month 48 (After intervention)
**Attention**		
Digit span D (digit sequence length) Digit span I (digit sequence length) Stroop test * I II III Trail making test A * Trail making test B * Digit symbol (WAIS-R) Figure completion (WAIS-R)	7 3 36" ↓ (0 errors) = 26" ↓ (0 errors) = 181" ↓↓ (14 errors) ↓↓ 679" ↓↓ Unable to complete 4 ↓↓ 7 =↓	6 4 20" ↓ (0 errors) = 23" ↓ (4 errors) ↓↓ 35" ↓ (18 errors) ↓↓ 80" ↓↓ Unable to complete 9 = 9 =
**Construction**		
Rey complex figure* copy Clock drawing* (max 10)	25 ↓↓ 5 ↓↓	32 ↓ 8 ↓
**Memory**		
Logical memory I (WMS-R) Logical memory II (WMS-R) Visual reprod. I (WMS-R) Visual reprod. II (WMS-R) Rey complex figure* imed	3 ↓↓ 0 ↓↓ 18 ↓ 0 ↓↓ 0 ↓↓	6 ↓↓ 0 ↓↓ 23 = 0 ↓↓ 0 ↓↓
**Language**		
Fluency (F.A.S.)* Fluency (Animals)* Boston naming test*	2 ↓↓ 1 ↓↓ 21 ↓↓	12 ↓ 12 = 55 =
**WCST**		
Categories achieved Set loss Perseverative err. Wechsler intelligence scales Digit span Information Similarities Comprehension Vocabulary Digit symbol Block design	Unable to engage in the task WAIS-R – scaled scores 8 = 9 = 11 = 9 = 8 = 4 ↓↓ 6 ↓	0 ↓↓ 0 ↓↓ 72 ↓↓ WAIS-III – scaled scores 11 = 8 = 14 ↑ 14 ↑ 10 = 9 = 11 =

= normal; ↑above average; ↓impaired; ↓↓severely impaired; numbers reported are raw scores, except for the WAIS-R and WAIS-III results, which are scaled scores, corrected by age, using the original norms of the tests;

* reference scores from the Compendium of Neuropsychological Tests^10^; WMS-R: Wechsler Memory Scale - Revised^11^; WAIS-R: Wechsler Adult Intelligence Scale - Revised^12^; WAIS-III: Wechsler Adult Intelligence Scale - III^13^; WCST: Wisconsin Card Sorting Test^14^.

**Table 2:** Functional Assessment pre and post intervention, accomplished through clinical interviews and behavioural observation.

	Month 10 (Before intervention)	Month 48 (After intervention)
**Orientation** **and memory**	• Could not tell current year, month or day • When in medical settings, used to say he was in a work meeting • Used to get lost in his own house • He had no record of his brain injury. If asked, he would have "no complaints" about health or cognitive abilities • Could not remember the youngest son, mistaking him for his brother • Could not give current address, phone number or city where he lived • Usually confused when asked about past autobiographical events	• Consistently knows current year and month • Usually knows where he is, and if not, looks for environmental clues • Finds his way around at home • He is able to report he had an aneurism and has memory problems • Remembers youngest son and usually knows what happened to who (e.g.: their wives´ names, or which son has had a baby) • Knows his address and phone number, and when in doubt, knows he has this information in his wallet • He is able to recall past information on his life more accurately
		
Initiation	• Would never seek or ask for food, drink or to use the bathroom • Could not initiate goal-oriented activities • Could not initiate conversations or spontaneous comments • He was able to follow simple verbal orders and answer direct questions	• Goes to the bathroom by himself when he needs to • He is able to prepare a cold drink or get cookies when he wishes • Chooses TV shows or DVD he wants to watch • Goes to bed by himself when he wants to sleep • He still does not start conversations, but comments appropriately on subjects being discussed and interacts more in family gatherings
		
Self monitoring	• Could not engage his attention on one task for more than a few minutes • Used to randomly walk, open and close doors and cabinets, and shake hands with anyone who drew close • Showed utilization behavior towards objects • Had physical aggression episodes with family and caregiver	• He stays focused on one task for longer periods, being able to go to the movies, follow a play and participate in collective games (cards, bingo) • He almost never shows aggression or inappropriate behaviors, except for getting a little restless when he is extremely tired

### Rehabilitation process

The neuropsychological intervention for MM was carried out through three equally important approaches:

- professionals working individually with the patient (twice a week): Our work intended to improve basic skills, such as focused attention, retaining information in the mind for longer periods, and verbal output, allowing MM to benefit more from the structure provided for him.
- professionals discussing problems and strategies with family and / or caregiver (once a week): We were able to provide theoretically based explanations for the problems seen in daily life, to devise possible interventions along with the family, and to set realistic goals for the patient in the time.
- family and caregiver applying strategies with patient at home: MM was not able to use compensatory strategies or deal with his schedule by himself. However, with training in his environment, he was able to carry out several tasks at home.

Main issues discussed with family and caregiver:

**Physical agression:** There were several episodes when MM had hit a family member or caregiver. The rehabilitation team helped the family not to interpret this behavior as a personal deliberate aggressive act, by discussing the failure of inhibition mechanisms, possible triggers for this kind of behavior such as confusion or tiredness, and ways to avoid these situations.

**Emotional expression:** Because of MM´s lack of initiation and spontaneity, he seemed “stern” most of the time, which was not necessarily an expression of sadness or bad mood. However, he was very dependent on the surroundings, so if one started asking if he was sad or worried, he would probably agree, start crying and “get into the mood” that was being somehow indicated. This generated a lot of worry in the family, and was addressed by discussing emotional regulation mechanisms and showing evidence that he also responded to “positive” questioning in the same way (e.g. “Are you happy to be here?”, “Are you enjoying this?”).

**Weekly schedule:** Having a fixed schedule of meaningful activities would help MM to be less disoriented, to regulate sleeping hours and to stabilize mood. Chosen activities should represent previous life and be compatible with MM´s present abilities. Patient should be encouraged to make decisions about his activities as frequently as possible.

**Environmental changes:** Since MM used to walk while looking down, placing signs on the doors and walls was not effective in helping him find the bathroom and his bedroom, and had him wandering around at night waking everyone up. Therefore, based on the principle of errorless learning (avoiding mistakes during the learning process) his wife developed a signaling strategy that blocked the doorways and made him read signs posted lower, directly at his eye level. Consequently, he was forced to stop and decide which way he was going, and then to head in the correct direction before unblocking the doorways, instead of randomly entering the rooms.

Main targets in the individual work with the patient:

**Orientation and memory:** Basically MM had to re-learn his life experiences through repetition, as if his story was being retold to him, registered in a diary and photo albums. This material was developed together with family and constantly used in several environments. Our main goal was help him store his autobiographical data in a semantic storage system, so he could use information from his personal history (e.g. “I have a younger son”) even without remembering specific events.

**Attention and self-monitoring:** Through meaningful tasks, ranging from very simple to more complex (e.g. reading a paragraph and finding a specific answer; going to a store near the clinic and choosing a gift for his wife), MM was encouraged to establish control over his mental processes, being focused on tasks with increasing duration and complexity.

**Verbal output:** MM was constantly involved in conversations about subjects he was highly familiar with. He was encouraged to explain concepts and ideas to others, and to also give his personal opinion. A direct result of this consistent effort could be seen afterwards at home, and at social gatherings.

## Discussion

We described the outcomes of neuropsychological rehabilitation in a patient with severe cognitive deficits due to the rupture of an ACoA aneurysm. The process was goal oriented and largely based on discussions with the family and caregiver.

The patient’s difficulties were primarily the result of his cognitive deficits in memory and executive function. Data on neuropsychological assessment pre and post intervention showed an improvement on some tests. Nevertheless, the impairment present, compared to the normal population, remained on the second evaluation for most of the tests. Nevertheless, the increase in some tests scores, although modest, should be seen as some improvement of function. Since the test contents were not directly trained on, the results shown do not reflect practice effects.

However, discussions in rehabilitation literature make clear that improvement on tests should not be the outcome of interest. According to Wilson,^15^ neuropsychological tests are important in identifying cognitive strengths and weaknesses, but the goal of rehabilitation is not to improve standardized cognitive test scores. Instead, rehabilitation should concentrate on the real-life problems that stem from neuropsychological deficits. Therefore, more attention should be paid to functional improvements.

At the first functional evaluation, the patient was severely disoriented, dependent for almost all daily activities, showing several behavioral disturbances. At the time of the second evaluation, significant improvements in most of these domains were observed. Hence, although the patient was not able to return to his pre-morbid activities, significant improvements were evident, resulting in better quality of life for both patient and family.

It is important to stress that MM´s family was especially involved and supportive, being very helpful during the whole process, which is not always the case. Other factors that could be related to the favorable outcome are the patient’s pre-morbid intellectual abilities and personality.^16^ Also, since the intervention proposed was not testing a specific technique, it is hard to ascertain how much of the improvement observed was due to natural recovery, medications, or rehabilitation efforts.

In sum, while much has been learned over recent years regarding the neurobehavioral and neuroanatomical sequelae of ACoA aneurysm rupture,^2-4^ studies addressing rehabilitation outcome of ACoA severe amnesics are extremely rare. We suggest that a neuropsychological rehabilitation approach focusing on goals based on the family and caregivers necessities is a sensitive and sensible manner in which to carry out cognitive rehabilitation in such severe cases. Although in some cases recovery is not as successful as to allow a full return to the patient’s premorbid activities, there is much which can be done for these patients and families, in terms of improvements in functional gain, emotional adjustment and quality of life.

## References

[1] Clinchot DM, Bogner JA, Kaplan PE (1997). Cerebral aneurysms: analysis of rehabilitation
outcomes. Arch Phys Med Rehabil.

[2] DeLuca J, Locker R (1996). Cognitive rehabilitation following anterior communicating artery
aneurysm bleeding: a case report. Disabil Rehabil.

[3] Simard S, Rouleau I, Brosseau J, Laframboise M, Bojanowskyc M (2003). Impact of executive dysfunctions on episodic memory abilities in
patients with ruptured aneurysm of the anterior communicating
artery. Brain Cogn.

[4] Johnson MK, O'Connor M, Cantor J (1997). Confabulation, Memory Deficits, and Frontal
Dysfunction. Brain Cogn.

[5] Parkin A J, Yeomans J, Bindschaedler C (1994). Further characterization of the executive memory impairment
following frontal lobe lesions. Brain Cogn.

[6] Böttger S, Prosiegel M, Steiger HJ, Yassouridis A (1998). Neurobehavioural disturbances, rehabilitation outcome, and lesion
site in patients after rupture and repair of anterior communicating artery
aneurysm. J Neurol Neurosurg Psychiatry.

[7] Beeckmans K, Vancoillie P, Michiels K (1998). Neuropsychological deficits in patients with an anterior
communicating artery syndrome: a multiple case study. Acta Neurol (Belg.).

[8] Akyuz M, Erylmaz M, Ozdemir C, Goksu E, Ucar T, Tuncer R (2005). Effect of temporary clipping on frontal lobe functions in
patients with ruptured aneurysm of the anterior communicating
artery. Acta Neurol Scand.

[9] Wilson BA, Evans JJ, Keohane C (2002). Cognitive rehabilitation: a goal-planning
approach. J Head Trauma Rehabil.

[10] Strauss E, Sherman EMS, Spreen O (2006). A Compendium of neuropsychological tests: Administration, norms, and
commentary.

[11] Wechsler D (1987). Manual for the Wechsler Memory Scale-Revised.

[12] Wechsler D (1981). WAIS-R Manual: Wechsler Adult Intelligence Scale-Revised.

[13] Wechsler D (1997). WAIS-III Manual: Wechsler Adult Intelligence Scale.

[14] Heaton RK, Chelune GJ, Talley JL, Kay GG, Curtiss G (1993). Wisconsin Card Sorting Test Manual.

[15] Wilson BA (2003). Neuropsychological Rehabilitation - Theory and Practice.

[16] Novack TA, Bush BA, Meythaler JM, Canupp K (2001). Outcome after traumatic brain injury: pathway analysis of
contributions from premorbid, injury severity, and recovery
variables. Arch Phys Med Rehabil.

